# Association Between Periodontitis and Preterm Birth in a Cohort of Pregnant Women in Ivory Coast

**DOI:** 10.3290/j.ohpd.b3464893

**Published:** 2022-10-19

**Authors:** Zocko Ange Désiré Pockpa, Assem Soueidan, Nadin Thérèse Koffi-Coulibaly, Gnaba Samson Mobio, Morgane Pere, Zahi Badran, Xavier Struillou

**Affiliations:** a Assistant Professor of Periodontology, Felix Houphouët Boigny University, Periodontology Department, Dental College, Abidjan, Côte d’Ivoire. Idea, study design, performed periodontal examinations, wrote the manuscript.; b Professor of Periodontology, CHU Nantes, Nantes Université, Periodontology Department, UIC Odontology 11, Nantes, France. Project supervisor, study design, synthesis of results, read and approved the manuscript.; c Assistant Professor of Periodontology, Felix Houphouët Boigny University, Periodontology Department, Dental College, Abidjan, Côte d’Ivoire. Project supervisor, study design, synthesis of results, read and approved the manuscript.; d Professor of Periodontology, Felix Houphouët Boigny University, Periodontology Department, Dental College, Abidjan, Côte d’Ivoire. Contributed to critical proofreading and discussion, read and approved the manuscript.; e Senior Engineer in Statistics, Nantes University Hospital, Research Department, Methodology and Biostatistics Platform, Nantes, France. Study design, statistical analysis; f Professor of Periodontology, College of Dental Medicine, University of Sharjah, Sharjah, UAE. Contributed to critical proofreading and discussion, read and approved the manuscript.; g Associate Professor of Periodontology, CHU Nantes, Nantes Université, Periodontology Department, UIC Odontology 11, Nantes, France. Study design, synthesis of results, read and approved the manuscript.

**Keywords:** adverse pregnancy outcomes, periodontal medicine, periodontitis, pregnancy, preterm birth

## Abstract

**Purpose:**

The aim of this study was to investigate the possible association between periodontitis and preterm birth in Ivory Coast.

**Materials and Methods:**

A cohort study including 446 volunteers (pregnant women) aged 15–50 years was performed in the Gynecology-Obstetrics Department of the University Hospital Center of Cocody-Abidjan in Ivory Coast. Socioeconomic and periodontal status was obtained during pregnancy. After delivery, obstetric data was collected. Periodontitis was diagnosed according to the new 2018 EFP/AAP classification of Periodontal and Peri-Implant Diseases and Conditions, as follows: a subject presenting with interdental CAL at two non-adjacent teeth or buccal/oral CAL ≥ 3 mm with pocketing > 3 mm was diagnosed with periodontitis. Any birth before the 37th week was considered a preterm birth (PTB).

**Results:**

The prevalence of periodontitis and preterm birth were 59.47% and 18.34%, respectively. Periodontitis was mainly stage 1. PTB was statistically significantly higher in pregnant women with periodontitis compared to women without periodontitis (p = 0.0002). Multivariate analysis showed that periodontitis was associated with PTB (p = 0.0002). Logistic regression showed that periodontitis is a risk factor for preterm birth (OR = 3.62; 95% CI: 1.80–7.31; p = 0.0003).

**Conclusion:**

The results of this study suggest that periodontitis is an additional risk factor for preterm birth in Ivory Coast.

Preterm birth (PTB) is any birth that occurs before the 37th week of amenorrhea of a fetus weighing more than 500 g at birth and breathing or showing any sign of life.^3^ PTB is the primary cause of neonatal morbidity and mortality in the world. PTB affects 11.1% of births each year.^2,3^ The main risk factors associated with PTB are alcohol, smoking, drug use during pregnancy, high or low maternal age (> 34 years old or < 17 years old), African-American ethnicity, low socioeconomic status, inadequate prenatal care, low maternal body mass index (BMI), high blood pressure, generalised/urogenital tract infections, cervical incompetence, diabetes mellitus, nutritional status, stress and multiple pregnancies.^2^ Despite the global effort to minimise these known risk factors, the worldwide prevalence of PTB remains high. Thus, other unknown risk factors could be related to the ongoing occurrence of PTB. Consequently, efforts to determine other causes of PTB continue.^3^

In 1996, Offenbacher^18^ first documented that periodontitis is a potential additional risk factor for PTB. Periodontitis is an immuno-inflammatory infection-induced condition affecting the tooth-supporting tissues.^20^ Without treatment, it can lead to tooth loss and negatively influence general health.^16,26^ Indeed, periodontitis has been associated with multiple systemic diseases, such as rheumatoid arthritis, diabetes, cardiovascular diseases and adverse pregnancy outcomes (APO).^11^ Since Offenbacher’s initial publication,^18^ several studies have been published on the relationship between periodontitis and obstetric outcomes.^7,13,21^ Most of these studies have confirmed the existence of an association between periodontitis and APO.^7,13,15,21^ Oral dysbiosis and inflammation appear to be central link of this association.^15,22,28,29^ Furthermore, it has been suggested that periodontal treatment would have a beneficial effect on obstetric outcomes.^15^

However, despite the increasingly convergent data in the literature on the existence of an association between periodontitis and obstetric outcomes, no study has been carried out in Ivory Coast. The aim of this study was to investigate the possible association between periodontitis and PTB in a cohort of pregnant women in Ivory Coast.

## Materials and Methods

### Study Design

The present prospective cohort study was designed to evaluate the association between periodontitis and PTB.

### Sample Size Calculation

Because data on the oral health of pregnant women in Ivory Coast were unavailable, a pilot study was performed for 45 days with 100 pregnant women. The prevalence of periodontitis was 67%. A sample size of 334 pregnant women was required, based on a periodontitis prevalence of 67% in pregnant women, and – setting the margin of error at 5% – a confidence level of 1.96. However, similar studies have reported 10% to 20% of participants being lost during follow-up.^4,22,23^ To compensate potential loss of participants, the initial sample comprised 509 pregnant women visiting prenatal care at the University Hospital of Cocody between January and December 2018. This study was approved by the scientific Research Ethics Committee of the Odonto-stomatology Faculty of Felix Houphouet Boigny University, Ivory Coast (protocol number 31/2018) and was conducted in accordance with the Helsinki Declaration of 1975, as revised in 2013. All subjects were informed about the aim of the study and provided their verbal and written consent before being included in the study.

### Study Population

**Fig 1:**
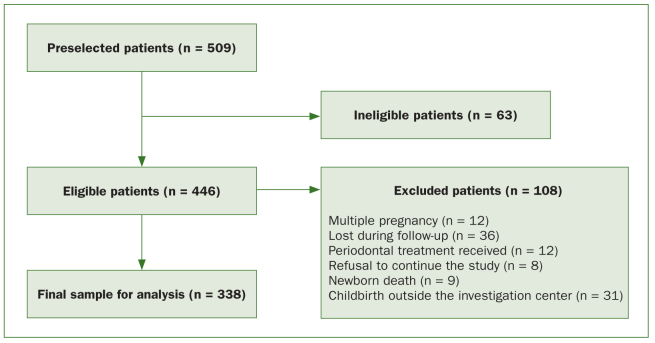
Sampling procedure.

The inclusion criteria to participate in the study were pregnant women with ≥ 6 teeth who had completed questionnaire and received the periodontal examination. The exclusion criteria were: periodontal treatment or antibiotics taken during the 3 months preceding the study. 446 women met the inclusion criteria. However, eligible women, previously included in the cohort, were withdrawn in the following situations: refusal to continue the study (8), multiple gestation (12), lost during follow-up (36), received periodontal treatment during follow-up by our study team (12), newborn death (9), or childbirth outside the investigation center (31). In total, data from 338 participants were analysed (Fig 1).

### Study Steps

After obtaining their written consent, all patients were interviewed with a standardised, structured questionnaire. This was followed by a periodontal clinical examination. After delivery, obstetric data were collected. The questionnaire was used to assess sociodemographic data (age, educational level, profession), obstetrics data (gestational age, date of first prenatal visit, primiparity, history of obstetric complications, method of delivery [vaginal or cesarean section]), lifestyle and oral hygiene habits (tobacco, alcohol, frequency of brushing, frequency of dental visits). The periodontal examinations were performed by a single examiner (PZAD) blinded to pregnancy outcomes using a Williams graduated periodontal probe (Michigan O probe, Hu-Friedy; Chicago, IL, USA) at six sites/tooth excluding third molars. Clinical measurements including the plaque index (PI), bleeding index on probing (BOP), probing depth (PD), periodontal recession and clinical attachment loss (CAL), were assessed at six sites per tooth for all teeth except third molars. PI was recorded by assigning a binary score to each surface (1 for plaque present, 0 for absent) and calculating the percentage of total tooth surfaces that revealed the presence of plaque detected using a disclosing agent. In the same way, full-mouth BOP was calculated after dichotomously assessing the presence of bleeding from the bottom of the pocket with a manual probe. Full-mouth PD and recession (REC) of the gingival margin were recorded at the same time, with measurements rounded to the nearest millimeter. REC was recorded as a positive value if the free gingival margin occurred apical to the cementoenamel junction (CEJ), whereas it was recoded as a negative value if it was coronal to the CEJ. Full-mouth CAL was calculated as PD plus REC. Obstetric data (gestational age, newborn weight, blood pressure, proteinuria, vaginal or cesarean delivery) were collected from birth registers by midwives who were blinded to the periodontal status of the participants.

### Definitions

#### Diagnostic criteria for periodontitis

According to the new 2018 EFP/AAP classification of Periodontal and Peri-Implant Diseases and Conditions, periodontitis was diagnosed as: interdental CAL at two non-adjacent teeth with buccal/oral CAL ≥ 3 mm, with pocketing > 3 mm.^19^ This classification is based on the stages and grades of periodontitis. In our study, only stages were considered. Periodontitis severity staging was defined by the interproximal CAL at sites with the greatest attachment loss: a CAL of 1–2 mm was defined as Stage I, 3–4 mm as Stage II and of ≥ 5 mm as Stages III–IV.

#### Diagnostic criterion for preterm birth

PTB was defined as a delivery at < 37 weeks of gestation (gestational age determined by last menstrual period and ultrasound fetal measurement).

### Statistical Analyses

All analyses were conducted using SPSS software version 22 (SPSS; Chicago, IL, USA) with a significance threshold set at p < 0.05. Continuous variables were expressed as mean ± standard deviation (SD) and Student’s t-test was used for comparison between groups. Categorical variables were expressed as numbers (n) and percentage (%), and the χ^2^ test was used for the comparison between the groups. Odds ratios (OR) were assessed by univariate logistic regression.

## Results

Out of the 509 initially examined women, 446 met the inclusion criteria. 108 of them (24.2%) were excluded. In total, a final sample of 338 women were analysed. Table 1 shows associations between gestational age and sociodemographic, obstetric, lifestyle, and oral-hygiene habit factors. PTB was not associated with sociodemographic and obstetric factors, but was rather associated with behavioural factors such as alcohol consumption (p = 0.03) and a low frequency of dental visits (p = 0.03).

**Table 1:** Association between gestational age and sociodemographic, obstetric, lifestyle and oral-hygiene habits variables

Variables	Total (n = 338; 100%)	PTB (n = 62; 18.3%)	NB (n = 276; 81.7%)	p-value
**Age in years**				0.6851
15–24	45 (13.31%)	10 (16.13%)	35 (12.68%)	
25–34	200 (59.17%)	34 (54.84%)	166 (60.14%)	
35–50	93 (27.51%)	18 (29.03%)	75 (27.17%)	
**Educational level**				0.1797
Upper secondary (university)	134 (39.64%)	23 (37.10%)	111 (40.22%)	
Lower secondary (middle school, high school)	100 (29.59%)	16 (25.81%)	84 (30.44%)	
Primary or less	104 (30.77%)	23 (37.10%)	81 (29.35%)	
**Stage of pregnancy**				0.357
1st trimester	38(11.24%)	9 (14.52%)	29(10.51%)	
2nd trimester	140(41.42%)	21 (33.87%)	119(43.12%)	
3rd trimester	160 (47.34%)	32 (51.61%)	128(46.38%)	
**1st prenatal visit**				0.3979
1st trimester	238 (70.41%)	39 (62.90%)	199 (72.10%)	
2nd trimester	77 (22.78%)	17 (27.42%)	60 (21.74%)	
3rd trimester	23 (6.81%)	6 (9.68%)	17 (6.16%)	
**Primiparous**				0.1783
Yes	106 (31.36%)	15 (24.19%)	91 (32.97%)	
No	232 (68.64%)	47 (75.81%)	185 (67.03%)	
**Alcohol consumption**				0.0360*
Yes	36 (10.65%)	2 (3.23%)	34 (12.32%)	
No	302 (89.35%)	60 (96.77%)	242 (87.68%)	
**Smoking**				1.0000
Yes	1 (0.30%)	0 (0.00%)	1 (0.36%)	
No	337 (99.70%)	62 (100%)	275 (99.64%)	
**Daily toothbrushing**				0.918
1 time	100 (29.59%)	17 (27.42%)	83 (30.07%)	
2 times	217 (64.20%)	41 (66.13%)	176 (63.77%)	
3 times or more	21 (6.21%)	4 (6.45%)	17 (6.16%)	
**Last dental visit**				0.031*
Never consulted a dentist	148 (43.79%)	30 (48.39%)	118 (42.75%)	
< 1 year before pregnancy	26 (7.70%)	3 (4.84%)	23 (8.33%)	
> 1 year before pregnancy	151 (44.67%)	23 (37.09	128 (46.38%)	
No idea	13 (3.85%)	6 (9.68 %)	7 (2.54%)	

PTB: preterm birth; NB: normal birth.

Table 2 lists periodontal data for all subjects grouped by gestational age (PTB group, normal-term group). A statistically significant association was found between PTB and oral hygiene level (p = 0.02) and gingival inflammation (p = 0.01). Women in the PTB group had statistically significantly deeper PD (6.8 mm ± 1.99 vs 5.5 ± 2.30; p = 0.00) and more severe CAL (2.29 ± 1.33 vs 1.52 ± 1.35; p = 0.00) compared to women who had a normal-term delivery. The prevalence of periodontitis and preterm birth were 59.5% and 18.3%, respectively. Periodontitis was mainly stage 1. A statistically significant association was found between PTB and periodontitis (p = 0.00).

**Table 2:** Association between gestational age and periodontal variables

Variables	Total (n = 338; 100%)	PTB (n = 62; 18.34%)	NB (n = 276; 81.66%)	p-value
**Oral hygiene**				0.0263*
Poor	245 (72.49%)	52 (83.87%)	193 (69.93%)	
Good	93 (27.51%)	10 (16.13%)	83 (30.07%)	
**Gingival inflammation**				0.0093*
Yes	213 (63.02%)	48 (77.42%)	165 (59.78%)	
No	125 (36.98%)	14 (22.58%)	111 (40.22%)	
Probing depth	5.78 ± 2.30	6.83 ± 1.99	5.54 ± 2.30	0.0001*
Clinical attachement loss	1.66 ± 1.38	2.29 ± 1.33	1.52 ± 1.35	0.0001*
**Presence of periodontitis**				0.0002*
Yes	201 (59.47%)	50 (80.65%)	151 (54.71%)	
No	137 (40.53%)	12 (19.35%)	125 (45.29%)	
**Severity of periodontitis**				< 0.0001*
Stage I	140 (41.43%)	23 (37.10%)	117 (42.39%)	
Stage II	38 (11.24%)	18 (29.03%)	23 (8.33%)	
Stage III /IV	23 (6.80%)	9 (14.52%)	14 (5.07%)	

*Statistically significant.

Table 3 shows the results of logistic regression analyses. After adjusting for confounding variables, logistic regression showed that periodontitis is a risk factor for preterm birth (OR = 3.62; 95% CI: 1.80–7.31; p = 0.00).

**Table 3:** Logistic regression

Variable	PTB	
	Odds ratio (CI 95%)*	p-value
Presence of periodontitis	3.62 [1.80–7.31]	0.0003*
Severity of periodontitis		< 0.0001*
Stage I	2.04 [0.95–4.39]	0.0686
Stage II	8.4 [3.35–21.06]	< 0.0001*
Stage III/IV	7.35 [2.36–22.89]	0.0006*

## Discussion

To the best of our knowledge, this study represents the first report on the association between periodontitis and adverse pregnancy outcomes in an Ivory Coast population. This study highlighted a statistically significant association between periodontitis and PTB. These results are in line with findings of some previously published cohort studies which demonstrated statistically significant associations, more or less strong, between the presence of periodontitis and the occurrence of premature deliveries.^19^ However, other cohort studies did not find a statistically significant association between PD and PTB.^19^ The discrepancies among these results may result from methodological heterogeneity of adopted protocols and definitions.^8,13,15,17^ Studies varied in sample size, type of population, elimination of confounding factors, and definition of periodontitis. Hence, the comparison of the different data from published studies is challenging.^12-14,25,26^ In the present study, the 2018 EFP/AAP classification of Periodontal and Peri-Implant Diseases and Conditions was used to determine the periodontal status.^19^ In the study by Agueda et al,^1^ periodontitis was defined as the presence of at least 4 teeth with at least 1 site with PP ≥ 4 mm + CAL ≥ 3 mm on the same site. In the Cissé et al^6^ study, periodontitis was diagnosed as the presence of at least 2 sites with PP ≥ 4 mm + CAL ≥ 3 mm. In the study by Rakoto-Alson et al,^23^ the diagnosis of periodontitis was confirmed in the presence of at least three sites on different teeth with CAL ≥ 4 mm. This lack of standardisation of the measurement methods and diagnostic criteria for periodontitis limits the possibility of interpretation and comparison between studies. The introduction by consensus of the new classification (2018) of periodontitis should allow a harmonisation of the diagnosis and evaluation criteria of the periodontal status for future investigations.^5^

In short, within the limits of this study, our results confirm the existence of an association between periodontitis and PTB in Ivory Coast. The risk of having PTB is 3.62-fold higher in the presence of periodontitis in pregnant women. Hence, we suggest the establishment of a prenatal oral consultation and the intensification of awareness programmes among pregnant women and health professionals on the interrelation between periodontal diseases and pregnancy. We therefore recommend that collaboration be strengthened between dental surgeons, midwives, gynecologists and obstetricians in order to optimise the care of pregnant women.

## Conclusion

Preterm births remain a real public health concern. In order to reduce their impact, emphasis is placed on managing known risk factors and searching for factors that are still unknown. Our study demonstrated a significant association between periodontitis and premature deliveries and suggest periodontitis as an additional risk factor for preterm birth in Ivory Coast. The results of this cross-sectional study will serve to strengthen awareness among health authorities, pregnant women, health professionals and the population.
